# A Rare Giant Cell Tumour in the Distal Radius of a Seven-Year-Old Girl: A Case Report

**DOI:** 10.7759/cureus.40270

**Published:** 2023-06-11

**Authors:** Somit Sarkar, Jayanta K Laik, Ravi Kaushal, Minakshi Mishra, Manoj Rajak

**Affiliations:** 1 Joint Replacement and Orthopedics, Tata Main Hospital, Jamshedpur, IND; 2 Orthopedics, Manipal Tata Medical College, Jamshedpur, IND; 3 Pathology, Tata Main Hospital, Jamshedpur, IND

**Keywords:** fibular graft, curettage, gct, skeletally immature, surgery for gct, bone graft, free fibula flap, wrist, giant-cell tumor

## Abstract

A giant cell tumour (GCT) is a benign and locally aggressive tumour that is usually observable in a skeletally mature patient involving the end of long bones. The reported incidence of this tumour in a skeletally immature patient is extremely rare. However, we report one such case in the distal radius of a seven-year-old female patient.

Having presented with painful swelling of the right distal forearm, she underwent clinical and radiological examination, and a diagnosis of distal radius GCT was made. The tumour was treated with curettage, fibular graft, and synthetic bone graft.

This case report shows the importance of including GCT in children as a differential diagnosis. This tumour may have a good prognosis if diagnosed and treated early.

## Introduction

A giant cell tumour (GCT) is considered benign, but it sometimes behaves as a locally aggressive tumour [1]. This was the initial definition and classification by Jaffe and Lichtenstein [1], but in 2020, the World Health Organization redefined and reclassified GCT from benign to intermediate group [2]. GCT accounts for 20% of all benign bone tumours and 5% of all primary bone tumours. It is common in the 20-40-year-old age group [3] and is more common in females, with the male-to-female ratio being 1:3 to 1:5 [4]. GCT is very rare before skeletal maturity [5]. Its common sites are the distal femur, the proximal tibia, and the distal radius [4], and it generally occurs at the epiphyses of long bones. Although metaphyseal or diaphyseal involvement without epiphyseal extension is a rare entity [1], metaphysis being involved before the epiphysis in GCT has been reported [6,7]. This study presented a very rare case of distal radius GCT in a skeletally immature patient, which was managed with curettage, fibular graft, and synthetic bone graft substitute.

## Case presentation

A seven-year-old female presented to our hospital with pain and swelling in the left distal forearm that has been ongoing for a duration of two months and progressively increasing in size since the first month. There was no history of trauma.

**Figure 1:**
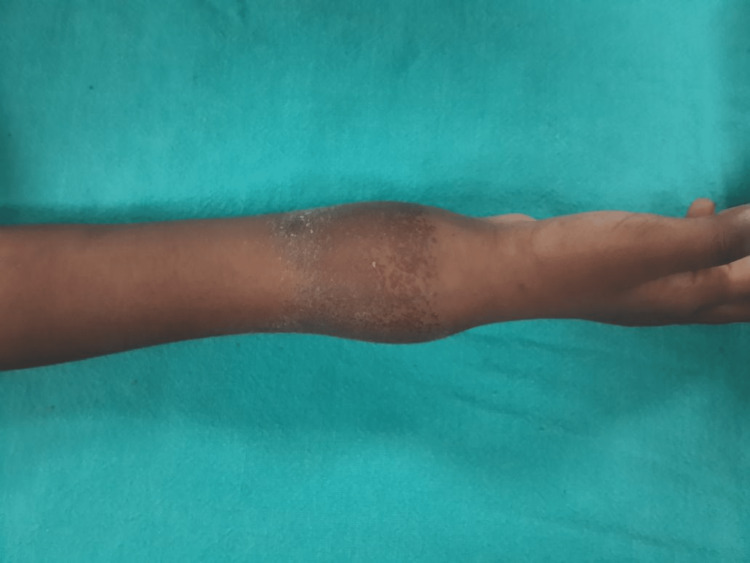
Clinical image of the wrist at presentation

Clinical examination revealed diffused swelling on the distal forearm (Figure 1).

**Figure 2:**
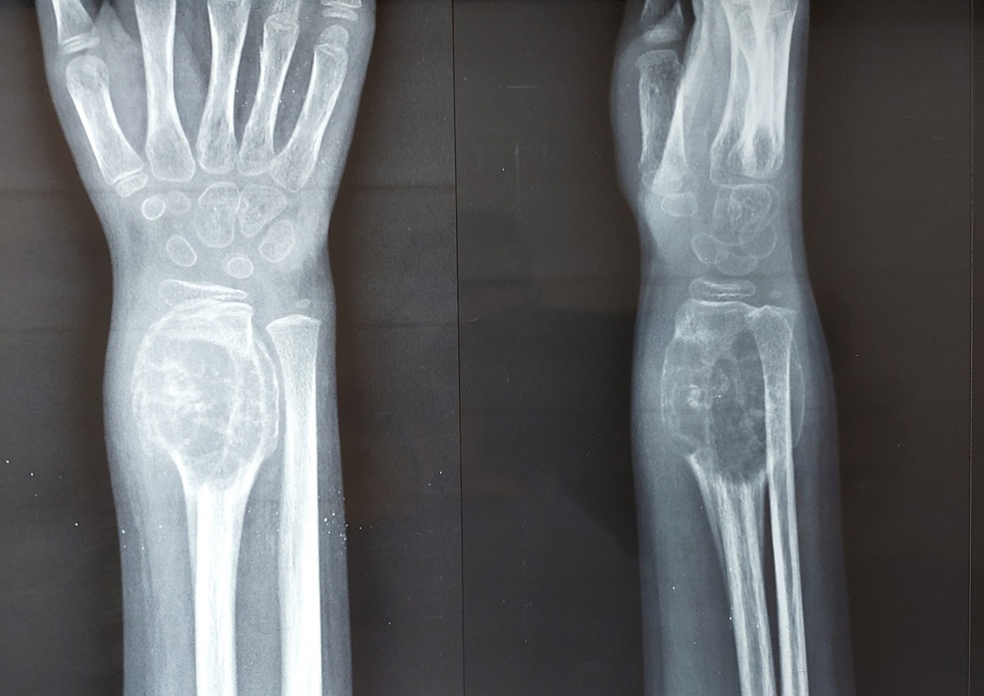
An X-ray at presentation showing an expansile lytic lesion at the distal end of the radius

The overlying skin was having patchy brownish pigmentation. On palpation, there was tenderness over the swelling, which was soft in consistency. Wrist movement was painful and restricted. However, there was no distal neurovascular deficit. An X-ray revealed an expansile lytic lesion with thinned-out cortices involving the meta-diaphyseal region without crossing the physis (Figure 2).

We decided to take a fine needle aspiration cytology of the bony lesion. The aspirate was a scanty blood-mixed material. The routine Papanicolaou and May-Grunwald Giemsa stain showed moderate cellular aspirate with fair numbers of a multinucleated, osteoclastic type of giant cells in discrete and small groups and oval to spindle uniform-looking stromal cells amidst a hemorrhagic background which, cytologically, was suggestive of a giant cell-rich lesion favouring a GCT.

As the patient was seven years old and obtaining sufficient autologous bone graft was difficult in this age group, it was decided to use a fibular bone graft, along with a synthetic bone graft substitute if the need arises. Counseling of the patient's relatives was done, and the operative procedure was explained.

**Figure 3:**
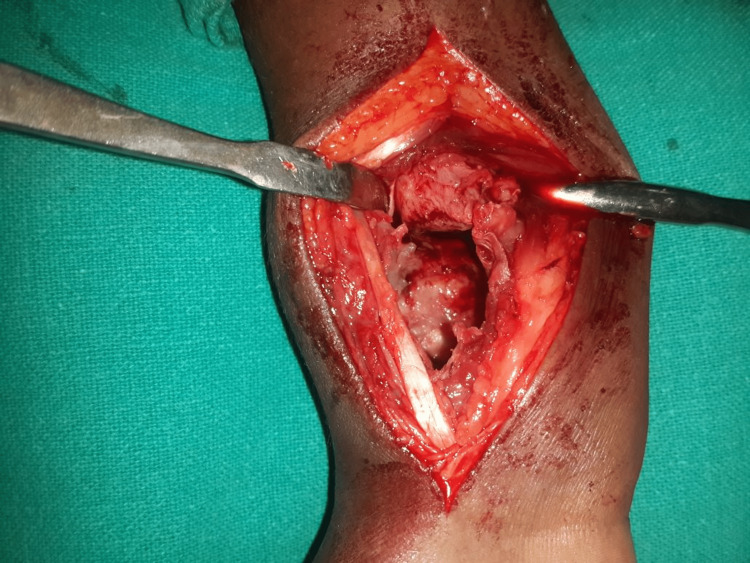
Intraoperative image showing the cavitary defect in the bone

Definitive surgery was performed through a volar approach of the distal forearm, exposing the tumour (Figure 3).

**Figure 4:**
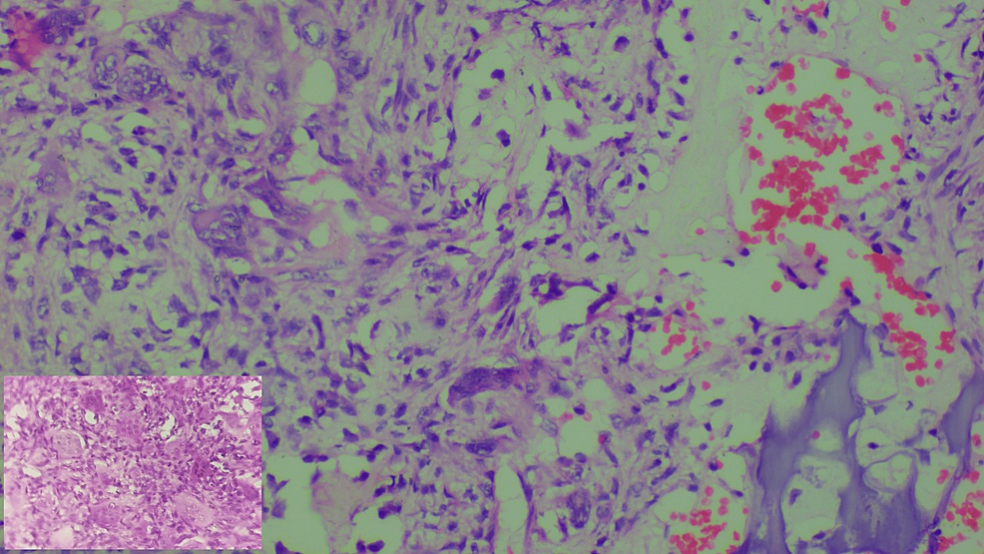
H&E X 200 - Microphotograph showing a giant cell-rich tumour comprising several multinucleated osteoclast-like giant cells and oval to spindle-shaped stromal cells, congested blood vessels, and tiny fragments of the cortical bone. Inset - H&E 10 X 10 - Microphotograph - Cluster of giant cells

After, the soft tissue dissection tumour wall was identified and the specimen was taken for histopathological examination (Figure 4).

**Figure 5:**
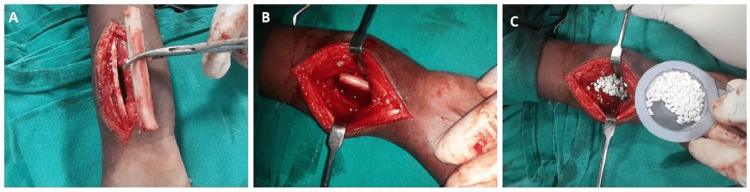
Intraoperative image showing (A) the harvested fibular strut graft, (B) the fibular strut graft applied in the defect, and the (C) filling of the defect with synthetic bone graft substitute in addition to fibular strut graft

Curettage was performed, and the resultant cavity was treated with hydrogen peroxide. Ipsilateral fibular graft with the approximate size of the cavity and a synthetic bone graft substitute were taken, filling the cavity (Figure 5).

**Figure 6:**
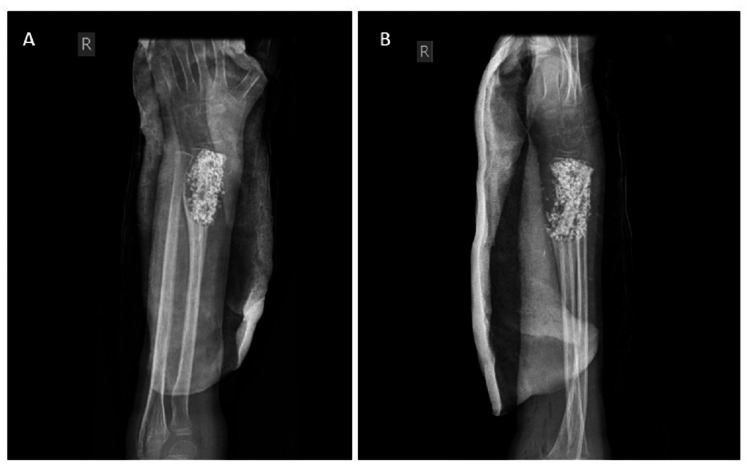
Immediate postoperative check X-ray: (A) AP view showing cavity filled with fibular strut graft and synthetic bone graft and the articular surface is intact and (B) lateral view showing the grafts without any articular surface breach

Then, closure was done in layers, as well well as dressing. A below-elbow plaster of the Paris slab was applied (Figure 6).

**Figure 7:**
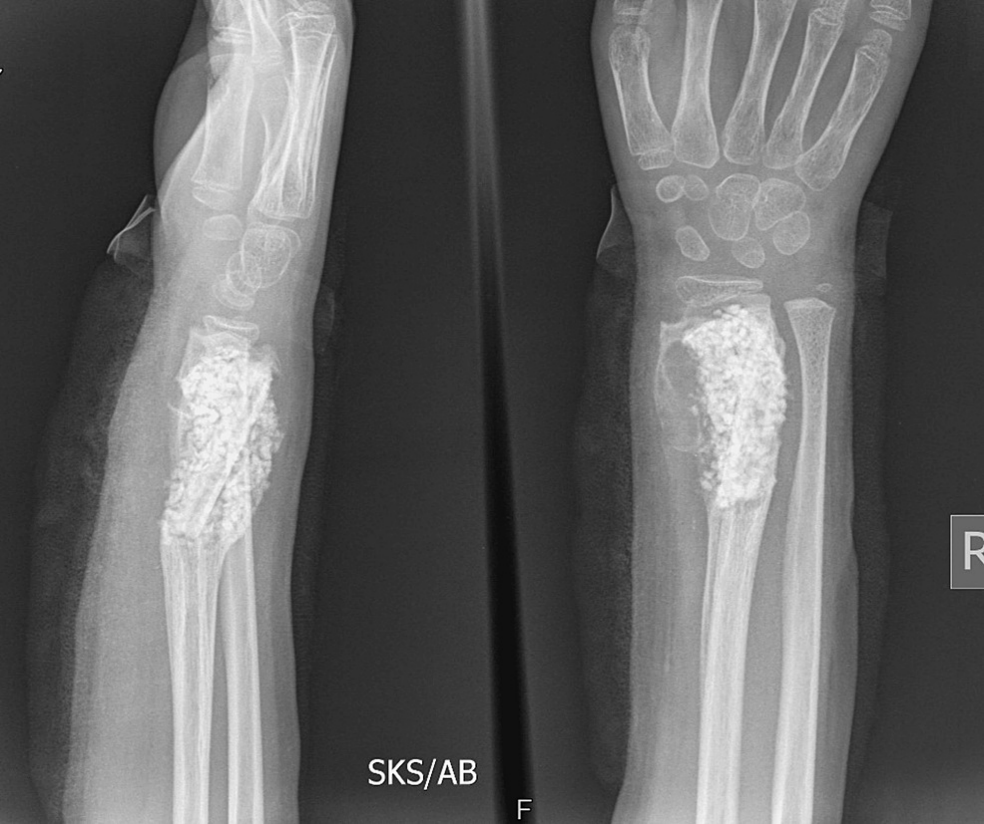
Check X-ray at the end of two months showing the incorporation of both fibular strut graft and synthetic bone graft

**Figure 8:**
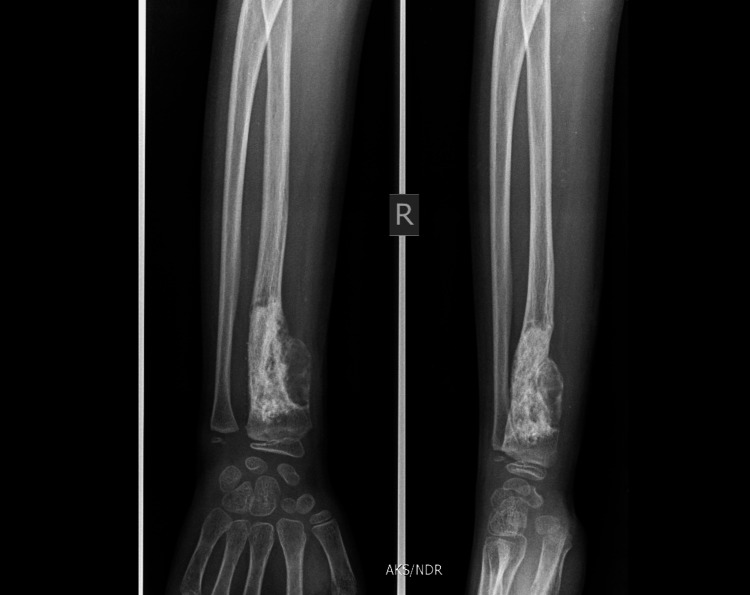
Check X-ray at the end of six months showing a good integration of the graft with the parent bone with obliteration of the cavity

**Figure 9:**
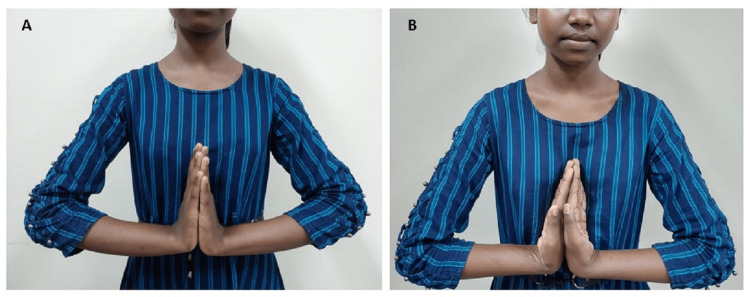
Functional outcome at the end of four years showing (A) a complete dorsiflexion and (B) a complete palmar flexion

**Figure 10:**
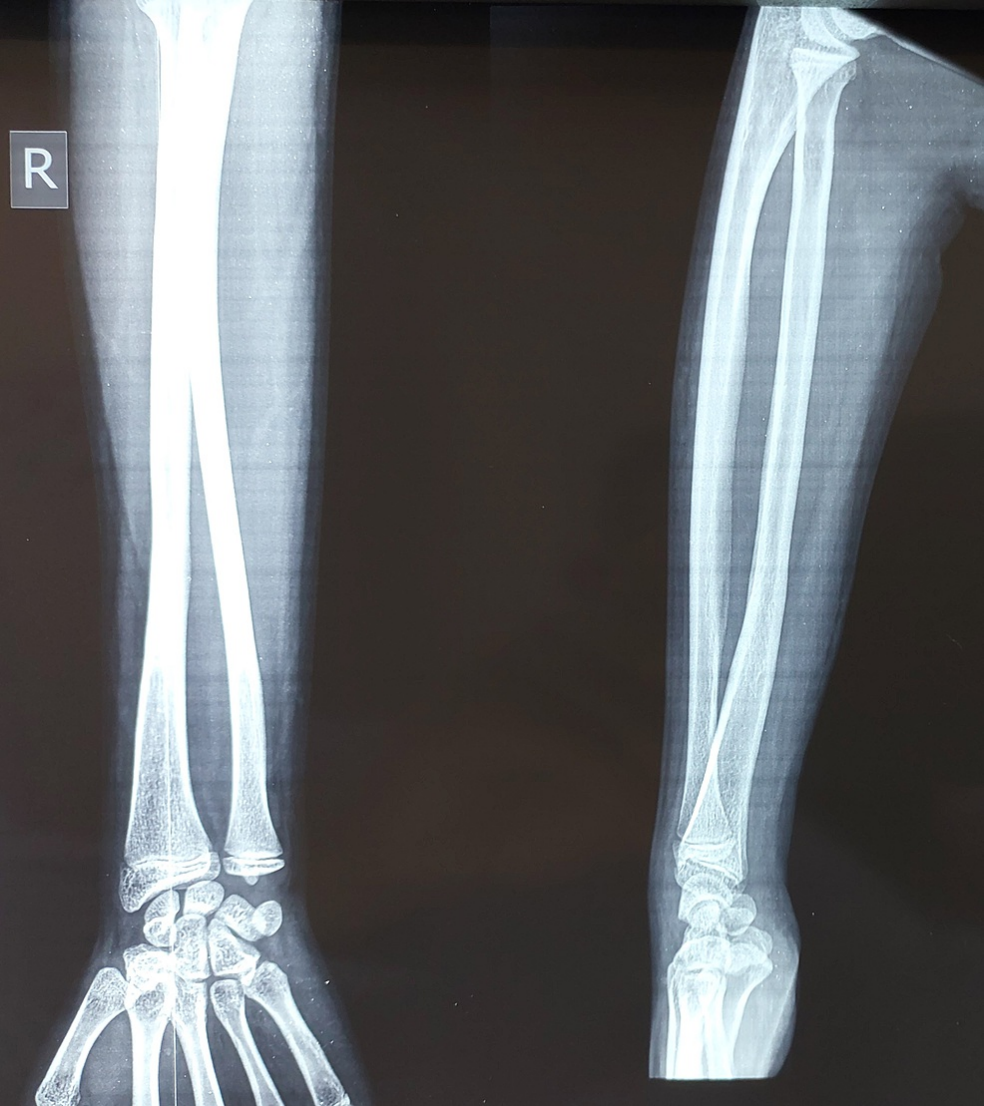
Final check X-ray at the end of four years showing complete radiological healing

Follow-up was done monthly for six months then yearly for four years (Figures 7, 8, 9, 10).

## Discussion

A GCT commonly occurs in the second to third decade of life. It is very rare in skeletally immature individuals with a reported incidence of only around 1.8% to 7.5% in the literature. A GCT has a slight female predominance and epiphyseal-metaphyseal location [8]. Some literature reported that open physis restricts GCT to the metaphyseal location while others reported otherwise that open physis does not restrict the GCT from penetrating the physis to become an epiphyseal-metaphyseal location [9]. A GCT usually occurs as a solitary lesion, and a multicentre occurrence is very uncommon, being reported in literature only around 1-2% [10]. The most common site of a GCT is around the knee (distal end femur and the upper end of the tibia) followed by the distal radius and the proximal humerus [4,7]. A GCT of the distal radius is known to exhibit highly aggressive behaviour with a high recurrence rate. The fact remains that histological examination is the gold standard in diagnosing GCT, which shows a giant cell-rich lesion, comprising of multinucleated, osteoclastic type of giant cells and cellular stroma with uniformly looking oval to spindle cells without any cellular pleomorphism, atypical mitosis or necrosis. Fragments of bone destruction and reactive bone may be seen [11,12].

In the paediatric age group, the goals of the treatment and management of a GCT are local control, maintaining joint function and preserving the physis. To illustrate, wide resection and reconstruction are applied in selected patients. Moreover, curettage and extended curettage of the lesion are employed to achieve local control and preserve joint function. This mode of treatment is used to treat lesions with Campanacci grade I, II, and even III with no joint invasion, with less than 50% metaphyseal destruction and uniplanar soft tissue mass [13]. Campanacci et al. [14] classified the GCT into three grades depending on their radiographic appearance: a grade 1 lesion (latent) has a well-defined margin and an intact cortex; a grade 2 lesion (active) has a relatively well-defined margin but no radiopaque rim, and the cortex is thinned and moderately expanded; and a grade 3 lesion (aggressive) has indistinct borders and cortical destruction. Wide resection and reconstruction is a treatment of choice in a select group of patients with Campanacci grade 3 lesions with joint invasion, multiplanar soft tissue component, and when the tumour covers a large area of subchondral bone [15].

Various treatment modalities for GCT are described in the literature, including curettage by itself; curettage and bone grafting; curettage and bone grafting in addition to adjuvant therapy such as phenol, liquid nitrogen, or hydrogen peroxide and coagulation using argon beam. All these treatment options have their own advantages and disadvantages [16]. Due to the variable recurrence rate of a GCT with different modalities, controversy still exists. It was noted by Bitoh et al. that a complete surgical excision can be curative for GCT [17]. Curettage and bone grafting are generally associated with good functional outcomes in most cases. However, as there are chances of recurrence in the utilization of curettage and bone grafting, much literature reported the use of polymethylmethacrylate (PMMA). PMMA generates heat, which is thought to cause thermal necrosis for the remaining tumour cells in the curetted cavity [18]. The benefit of using PMMA therapy include the absence of donor-site morbidity, abundant supply of the material, immediate structural stability, low cost, and ease to use [19]. Some articles reported a low recurrence of tumour and the early detection of recurrence after using PMMA as the cavity-filling agent. Few pieces of literature reported postoperative fracture after using extended curettage and cementing [7]. The drawbacks of using cement, however, include difficulty in its removal in recurring cases [20]. Several pieces of literature reported the high-speed burring technique of the cavity after simple intralesional curettage that causes thermal effect and the removal of residual tumour in most of the cases. This was followed by PMMA cementing and a bone graft or bone graft substitute [21].

Local recurrence [22], pathological fracture [23], and limited joint movement [24] are common complications. Lung metastasis and malignant transformation are other lesser-known complications. The literature reported pulmonary metastases in GCT from 1% to 6% [25]. The risk of lung metastasis increases by six times as reported after local recurrence of the disease and especially in grade III lesions [1]. Local recurrence with metastasis can be managed by further curettage and with the use of PMMA cementing, with a slight risk of increased morbidity [26].

We found very few reports of GCT in skeletally immature patients [5,8,27,28]. In our case, a joint was not involved; hence, curettage and ipsilateral fibular strut graft along with bone graft substitute was used. The lesion healed well without any recurrence with good functional outcomes after four years of follow-up.

## Conclusions

Most of the reported cases of GCT in literature are observed in patients in their late teens (13 to 18 years). However, GCT is rarely seen in patients who are less than 10 years of age. With the study presented, GCT should, hence, be considered as a differential diagnosis in this age group if clinical and radiological features are suggestive of the tumour. Moreover, histopathological examination is the most important diagnostic tool in skeletally immature patients, and early diagnosis and treatment result in a good and functional outcome.
